# Out-of-pocket payments for treatment of COVID-19 in Iran

**DOI:** 10.1186/s12962-022-00350-7

**Published:** 2022-03-19

**Authors:** Ali Reza Yusefi, Gholamhossein Mehralian, Abdolvahed Khodamoradi, Roghaye Abbasi, Fatemeh Vatankhah, Fatemeh Heaidari, Peivand Bastani

**Affiliations:** 1 Department of Public Health, School of Health, Jiroft University of Medical Sciences, Jiroft, Iran; 2 School of Pharmacy, Shahid Beheshti University of Medical Sciences, Tehran, Iran; 3 Department of Health Policy and Economics, Social Security Research Institute, Tehran, Iran; 4 Student Research Committee, School of Health, Jiroft University of Medical Sciences, Jiroft, Iran; 5 Faculty of Health and Behavioral Sciences, School of Dentistry, University of Queensland, Brisbane, QLD 4072 Australia

**Keywords:** Out-of-pocket payment, COVID-19, Coronavirus, Hospital

## Abstract

**Introduction:**

Out-of-pocket (OOP) is among the payment methods in Iran’s health system. The present study aimed to examine the OOP treatment costs for patients with COVID-19 in Iran.

**Methods:**

A descriptive-analytical, cross-sectional study was conducted in 2021. In this study, the cost records of 550 patients with COVID-19 hospitalized in a referral center of COVID-19 were selected using the stratified random sampling method. The required data were collected using a researcher-made questionnaire. Data were analyzed by t-test, ANOVA, and Pearson’s correlation coefficient in SPSS software version 23 at *p* = 0.05.

**Results:**

The total direct costs were 1,037,992.15 US $. Moreover, the shares of patients (OOP), basic insurance, government subsidy, supplementary insurance, discounts, and out-of-government subsidy in the total direct costs were US $ 92,231.21, 746,932.99 US $, 155,127.08 US $, 39,690.25 US $ and 4010.61 US $, respectively. In addition, the results confirmed that there was a positive and significant relationship between the patients’ OOP payments and the length of stay. It also found that the patients’ OOP payments are subject to the type of insurance program and discharge method.

**Conclusion:**

According to the results, 8.89% of the total direct costs were directly paid out of the patients’ pockets. The research findings confirm the urgent need to make decisions and implement effective interventions for COVID-19 disease by controlling risk factors and exploiting other countries’ successful experiences and international organizations’ recommendations to decrease the prevalence of the infected and consequently reduce the financial pressure of the disease on patients by approving the expansion of the insurance organizations’ role.

## Introduction

In late 2019, the COVID-19 pandemic and the subsequent rapid epidemic trend worldwide have raised concerns in different countries [1]. The virus’s rapid spread has left governments with prominent infected individuals [2]. Millions of individuals worldwide are now hospitalized because of the COVID-19 pandemic [3]. In Iran, the first case of COVID-19 was formally announced on February 20, 2019. Given the rapid transmission of this disease, the reported prevalence and prevalence rates seem to be of great concern [4]. According to the World Health Organization (WHO), by August 10, 2021, more than 200 million positive cases have been confirmed, and more than four million deaths from COVID-19 are estimated worldwide. In this regard, Iran accounted for more than four million cases and 95,000 deaths from COVID-19 [5]. On this date, the daily incidence of new cases in Iran with 40,808 is ranked first among other countries [5].

In addition to stewardship, generating resources, and providing services, financing is one of the four main functions of the health system. The conventional techniques of financing health care services include taxes, social insurance, private health insurance, and out-of-pocket (OOP) payments [6]. If the government fails to finance health care services, the financial burden will be directly imposed on individuals who must have the OOP payment [7]. The direct cost paid by the household instead of a service is called OOP payment [8].

Although OOP is typical in both developed and developing countries, it is the most inefficient way of financing the health system and is a defective mechanism for risk aggregation [9]. OOP can have serious adverse effects, including access to health services, especially among the poor [10]. Increasing OOP for private and public health services has led many households towards poverty and intensified the poverty of the poor [11]. The OOP financing is often a declining payment method for health services, and many individuals are imposed by catastrophic costs [12]. With the high price of health care services, about 44 million households (above 150 million persons) worldwide face exorbitant costs annually [13]. Even with health insurance, patients’ OOP seems to increase the risk of poverty [14].

In low-income countries, lack of insurance poor coverage, and insufficient social support have raised the OOP payments for households [15]. In Asian countries, OOP payments are one of the main methods of financing the health system [16, 17]. In general, OOP accounts for 18.6% of total health system expenditures in the third world [18].

In addition to the physical and physiological burden, COVID-19 has imposed a substantial financial burden on the health systems in different countries. In a study on the economic burden of COVID-19 in China, Jin et al. reported the total health and social costs of COVID-19 to be 0.62 and 383.02 billion dollars, respectively [19]. In research on the consequences and economic burden of 173,942 patients with COVID-19 hospitalized in the United States, it is estimated that the average hospitalization cost is $ 12,046 [20]. Another research in the US conducted on a large sample of 4075 patients to assess the OOP for COVID-19 found that Medicare advantage patients and privately insured patients contributed to hospitalization costs 49.1% and 71.21%, respectively. In this study, the average OOP for both groups was about 2688$ [21].

Measuring and monitoring health expenditures would help the policymakers in the health system select appropriate policies to protect patients [22]. Households’ OOP rate and the consequent incurrence of exorbitant health care costs are two main factors to be considered in health care planning and policy-making [23]. Given the significance of OOP and its continuous evaluation to monitor justice in financing the health system, this study evaluated the OOP rate among patients with COVID-19 admitted to the referral hospital for treatment of COVID19. Therefore, the main research question in this study is how much the OOP is in patients with COVID19 hospitalized in the public sector.

## The study setting

Financing the health system in Iran is underpinned by multiple models encompassing the public budget, social, private, supplementary insurance, and OOP. According to Iran’s National Health Accounts, OOP is usually above 50% and ranges from 50.4% in 2004 to 52% in 2013 [24–26]. In Gharibi (2013) and Keshavarz’ (2012) studies during 2011–2013, the OOP rates were 55 and 59.7%, respectively [27, 28]. Moreover, Amery (2013) and Kavosi (2009) revealed that the exorbitant costs were 8.3 and 14.2%, respectively [29, 30]. Hajizadeh and Nghiem (2011) considered the OOP rate above 50% in Iran as one barrier to access health services [31].

The Iranian health system has faced quite a few challenges in terms of financing and the provision of healthcare, so much so that 3% of the people were faced with catastrophic health expenditure annually, leading to dissatisfaction among the citizens [32]. As a practical solution, within the last four decades, the Iranian health system has been initiating several reforms to pave the way towards universal health coverage (UHC) such as the establishment of an extensive Primary Health Care (PHC) network, the family physician program, and recently Health Transformation Plan (HTP) [33]. UHC that assures access to necessary health care services by all the population needs sustainable financing [34]. However, inefficient stewardship of the Iranian healthcare systems and the weak political support for investing in the healthcare sector, on the one hand, and the existence of the unfair international sanctions, on the other hand, can prevent the inflow of financial flows to Iran and consequently, it is hard to achieve the UHC [35]. That's why out-of-pocket payment is highlighted more than the other sustainable and fair financial resources.

According to a report by the WHO in 2000, countries and health policymakers were encouraged to provide equitable funding [36]. To this end, reforms in the health sector have been of interest to all health policymakers over the past decade, particularly in developing countries [37]. In this regard, the Ministry of Health in Iran implemented the health system transformation plan, which adopted three approaches (namely protecting individuals financially, creating justice in access to health services, and improving the quality of services in hospitals since May 2014. This plan was a stepping stone toward achieving all the ideals of the health system, especially the financial protection of patients, by providing basic health insurance for the uninsured and decreasing the payments for patients admitted to hospitals affiliated with the Ministry of Health [37]. The implementation of this plan aimed to protect citizens financially against health costs. According to the goal set in this plan, the OOP rate for inpatient services in public hospitals should be decreased by < 10% [38].

## Methods

**Fig. 1:**
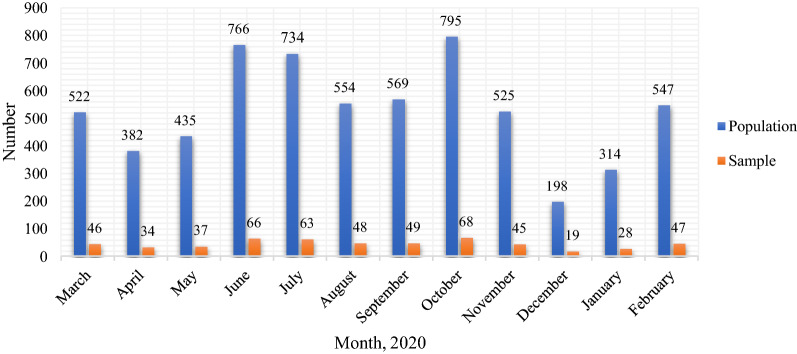
Population and a statistical sample of patients with COVID-19 admitted to the Hospital

A cross-sectional descriptive-analytic study was conducted in 2021. The study’s statistical population encompassed the financial records of patients with COVID-19 hospitalized in a referral hospital in Shiraz, as COVID-19 Reference Hospital in southern Iran from April to late March 2020. Considering the population of patients with COVID-19 in 2021 (i.e., N = 6341) and the following formula, the sample size was 550 hospitalized patients. By dividing 550 by 6341 and multiplying the obtained number by the number of patients admitted per month, the desired sample size was obtained per month (stratified sampling proportional to the size). The random sampling method was adopted to select the sample based on patients' file numbers and the table of random numbers (Table 1, Fig. 1).

$$n = \frac{{Nz^{2} pq}}{{Nd^{2} + z^{2} pq}}$$

where, N = 6341; p = q = 0.5; z = 1.96; d = 0.04.

**Table 1:** Population and a statistical sample of patients with COVID-19 admitted to the hospital

Row	Month	Population	Sample
1	March	522	46
2	April	382	34
3	May	435	37
4	June	766	66
5	July	734	63
6	August	554	48
7	September	569	49
8	October	795	68
9	November	525	45
10	December	198	19
11	January	314	28
12	February	547	47
–	Total	6341	550

The data collection instrument was a researcher-made three-section form. The first section of the form addressed the patients' demographic information (age, gender, marital status, length of stay, discharge method, and type of insurance). The second and third sections dealt with the frequency of services and expenditure information, respectively. The former section encompassed the number of visit services, counseling, rehabilitation, CT scans, radiography, laboratory, dialysis, radiology, ultrasound, and echo. Expenditure information addressed total direct costs, patient share (OOP), basic insurance share, government subsidy, supplementary insurance share, discounts, and out of subsidy commitments from total direct costs by type of health care services.. For observing research ethics, all parts of the questionnaire were anonymous, and data concerning the costs have been kept confidential with one of researchers (ARY).

To collect the required data, after obtaining the approval of the Shiraz University of Medical Sciences, the researchers referred to the hospital's accounting unit, and the patients’ bills were received from the hospital’s health information system (HIS). Considering the ethical principles, all data collection forms were completed without mentioning patients’ names. After the data collection procedure, the data were analyzed using descriptive and inferential statistical methods, T-test, ANOVA, Pearson's correlation coefficient, and multiple linear regression with SPSS software version 23 at p = 0.05. We performed Pearson’s correlation to test the relationship between the OOP expenses with patients' age and the length of stay. T-test has been used to investigate the mean difference between the OOP expenses based on patients' gender and marital status. The ANOVA test has been applied to analyze any differences between the OOP expenses and variables such as type of insurance and discharge method. Further, we performed multiple linear regression to determine the simultaneous effect of background variables on the OOP expenses.

## Results

### Participants’ demographic information

The patients’ mean age was 56.29 ± 15.99 years, and most of the participants (23.09%) were in the age group of 51–60 years. Moreover, 54.2% of the patients were male, and most of the patients (93.1%) were married and had social security insurance (52.91%). The mean length of stay in the hospital for the patients was 6.64 ± 5.57 days, and most of these individuals (56.36%) were hospitalized for 5–10 days. Furthermore, 84.5% of the patients were discharged from the hospital in the “recovery” status. Table 2 shows the frequency distribution of the patients.

**Table 2:** Participants’ demographic information (n = 550)

Variable	Category	Number	Percentage
Gender	Female	252	45.8
	Male	298	54.2
Age (year)	< 20	2	0.37
	20–30	20	3.64
	31–40	82	14.91
	41–50	91	16.54
	51–60	127	23.09
	61–70	118	21.45
	> 70	110	20
Marital status	Single	38	6.9
	Marital	512	93.1
Length of stay	< 5	178	32.36
	5–10	310	56.36
	11–15	33	6
	16–20	15	2.73
	> 20	14	2.55
Type of insurance	Health Service	192	34.91
	Social security	291	52.91
	Armed Forces	51	9.27
	Other	16	2.91
Discharge	Recovery	465	84.5
	Death	50	9.1
	Transfer	28	5.1
	Voluntary clearance	7	1.3

### Frequency of services provided to participants

According to the results, the highest frequency of services provided to the patients was for “laboratory” services with > 50,000 tests (average 107.70 tests per patient) (Table 3).

**Table 3:** Frequency of services provided to patients (n = 550)

Type of service	Number	Mean	Standard deviation
Visit	4342	7.89	5.90
Counseling	433	0.79	1.05
Rehabilitation	524	0.95	3.56
CT scans	560	1.02	1.17
Radiography	1574	2.86	2.34
Laboratory	59,234	107.70	120.69
Dialysis	8	0.01	0.12
Radiology	182	0.33	1.20
Ultrasound	56	0.10	0.34
Echo	54	0.10	0.30
Hoteling (Regular bed)	2887	5.25	3.20
Hoteling (Special bed)	763	1.39	4.94
Other services (Transport, Food)	1326	2.41	1.93

### Direct costs of all patients

**Fig. 2:**
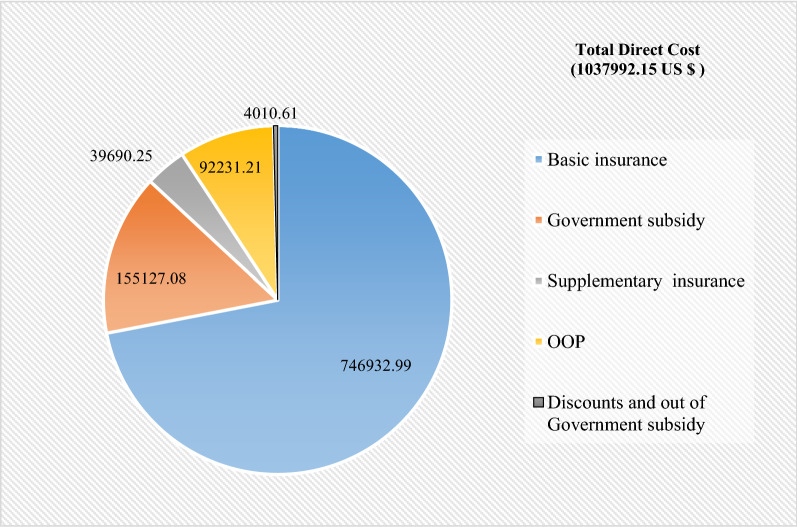
Shares of basic and supplementary insurance, government subsidy, patients (OOP), and discounts in the total direct cost of patients (US $)

The study results indicated that the total direct cost was 1,037,992.15 US $, with 71.96% basic insurance share (746,932.99 US $,), 14.94% government subsidy share (155,127.08 US $), 3.82% supplementary insurance share (39,690.25 US $), 8.89% share of patients (OOP) (92,231.21 US $), and 0.39% discounts and out of subsidy commitments (4010.61 US $) (Table 4 and Fig. 2).

**Table 4:** Shares of basic and supplementary insurance, government subsidy, patients (OOP), and discounts in the total direct cost of patients (US $)

Category	Amount	Mean	Standard deviation	Percentage of total direct costs
Basic insurance	746,932.99	1358.05	1910.03	71.96
Government subsidy	155,127.08	282.04	614.89	14.94
Supplementary insurance	39,690.25	72.16	348.69	3.82
OOP	92,231.21	167.69	271.21	8.89
Discounts and Government subsidy	4010.61	7.29	6.41	0.39
Total direct cost	1,037,992.15	1887.25	2562.55	100

^*^Values are expressed in US $

**Fig. 3:**
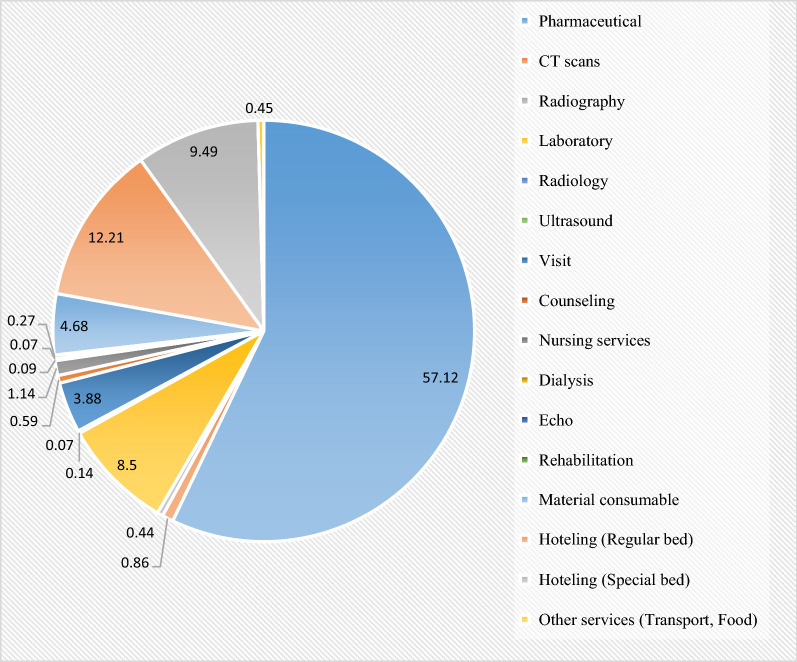
Patients’ OOP percentage by service type

The results indicated that among the types of services, pharmaceutical services with 45.09% of the total direct costs (466,249.91 US $) and 57.12% of the patients’ share (52,681.39 US $) had the highest shares in OOP (Tables 5, 6 and Fig. 3).

**Table 5:** Shares of basic and supplementary insurances, government subsidy, and patient in OOP by service type

Type of service	Total Amount	Total basic insurance	Total government subsidy	Total Supplementary insurance	OOP	Percentage of total direct cost
**Pharmaceutical**	466,249.91	247,798.40	133,512.24	31,269.61	52,681.39	45.09
**CT scans**	9319.98	8808.84	83.33	147.61	791.21	0.90
**Radiography**	4571.92	4162.26	13.51	64.88	402.38	0.44
**Laboratory**	80,625.58	67,429.97	6126.96	1356.29	7837.54	7.80
**Radiology**	1991.57	1739.82	128.92	12.93	127.36	0.19
**Ultrasound**	724.32	647.41	0	12.62	66.32	0.07
**Visit**	81,641.16	74,296.98	223.07	660.02	3580.52	7.89
**Counseling**	11,526.61	10,766.21	60.01	59.81	544.32	1.12
**Nursing services**	15,787.64	14,512.61	77.19	196.11	1055.37	1.53
**Dialysis**	2576.14	1957.40	180.45	4.98	86.09	0.25
**Echo**	1983.61	1819.93	6.53	24.52	62.67	0.19
**Rehabilitation**	4695.23	4365.43	45.53	44.77	244.45	0.45
**Material consumable**	28,340.71	9446.11	13,587.20	1658.83	4320.87	2.75
**Hoteling (Regular bed**	141,997.04	129,650.57	0	1488.76	11,260.55	13.73
**Hoteling (Special bed)**	169,856.59	158,927.37	0	2657.56	8755.83	16.43
**Other services (Transport, Food)**	12,093.46	10,603.62	1082.07	30.91	414.28	1.17
Total	1,033,981.55	746,932.99	155,127.08	39,690.25	92,231.21	100

*The total direct cost of services excluding "discounts and out of subsidy commitment”

**Values are expressed in US $

**Table 6:** The mean and standard deviation of basic and supplementary insurance, government subsidy, and patient shares in OOP by service type

Type of service	Mean (standard deviation)				
	Total amount	Basic insurance	Government subsidy	Supplementary insurance	OOP
Pharmaceutical	847.72 (1163.86)	450.54 (639.81)	242.75 (552.61)	56.85 (298.09)	95.78 (193.09)
CT scans	16.94 (18.01)	16.01 (25.86)	0.15 (1.49)	0.26 (1.29)	1.43 (3.49)
Radiography	8.31 (6.86)	7.56 (6.37)	0.02 (0.24)	0.11 (0.41)	0.73 (1.33)
Laboratory	146.59 (155.90)	122.61 (134.49)	11.13 (25.51)	2.46 (8.78)	14.25 (21.01)
Radiology	3.62 (31.42)	3.16 (24.15)	0.23 (4.43)	0.02 (0.22)	0.23 (3.06)
Ultrasound	1.31 (4.69)	1.17 (4.22)	0 (0)	0.02 (0.18)	0.12 (0.97)
Visit	148.43 (100.02)	135.08 (92.01)	0.41 (2.49)	1.21 (3.72)	6.51 (7.52)
Counseling	20.95 (28.49)	19.57 (32.69)	0.11 (1.23)	0.11 (0.49)	0.99 (2.46)
Nursing services	28.70 (48.64)	26.38 (45.25)	0.14 (1.53)	0.35 (1.49)	1.91 (3.56)
Dialysis	4.68 (35.04)	3.55 (27.86)	0.32 (7.56)	0.009 (0.21)	0.15 (2.26)
Echo	3.60 (11.10)	3.31 (10.18)	0.01 (0.09)	0.04 (0.31)	0.11 (0.47)
Rehabilitation	8.53 (34.21)	7.93 (31.72)	0.08 (1.13)	0.08 (0.66)	0.44 (2.11)
Material consumable	51.52 (114.28)	17.17 (48.82)	24.71 (64.75)	3.01 (13.24)	7.85 (22.24)
Hoteling (regular bed	258.17 (218.96)	235.72 (144.75)	0 (0)	2.71 (5.91)	20.47 (24.85)
Hoteling (special bed)	308.83 (1124.51)	288.95 (869.91)	0 (0)	4.83 (25.37)	15.92 (63.44)
Other services (transport, Food)	21.98 (25.38)	19.27 (34.81)	1.96 (1.01)	0.05 (0.52)	0.75 (2.71)

^*^Values are expressed in US $

### Relationship between demographic variables with total direct cost and OOP

Considering the results, there was a statistically significant relationship between the patients’ OOP payments and length of stay (r = 0.543, *P* < 0.001). As Table 7 shows that the patients’ OOP payments are subject to type of insurance program (F = 8.399, P < 0.001) and discharge method (F = 6.991, P < 0.001).

**Table 7:** Relationship between patients’ demographic variables with their total cost and OOP payments

Demographic variables	Main variables	Type of test and significance	
		Pearson’s correlation coefficient	P value
Age	OOP	0.052	0.225
	Total direct cost	0.092	0.03
Length of stay	OOP	0.543	< 0.001
	Total direct cost	0.888	< 0.001

Main variables		T-test (t)	P value
Gender	OOP	0.74	0.45
	Total direct cost	0.18	0.59
Marital status	OOP	0.93	0.34
	Total direct cost	1.12	0.26

Main variables		ANOVA (F)	P value
Discharge	OOP	6.991	< 0.001
	Total direct cost	27.44	< 0.001
Type of insurance	OOP	8.399	< 0.001
	Total direct cost	2.090	0.02

For post hoc analysis, the Scheffe test indicated that average OOP was significantly different among death and two main groups: recovery (P = 0.001) and transfer group (P = 0.004). As such, the average OOP was 167.21 and 229.97 units larger in the death group than the recovery and transfer group, respectively. Also, the average OOP was significantly different between patients covered by armed forces insurance and health services insurance (P = 0.009) and patients covered by armed forces insurance and social security insurance (P = 0.04). The average OOP in patients covered by armed forces insurance decreased by 197.41 units compared to patients covered by health services and 101.93 units compared to patients covered by social security insurance.

The multivariable regression of the OOP is first influenced by the length of stay, followed by the type of insurance program. Adjusted R-Squared was calculated at 0.29 (Table 8).

**Table 8:** Variables affecting OOP based on multiple linear regression

Variable definition	Variable	Unstandardized coefficients		Standardized coefficient β	t	P-value*
		B	Std. error			
–	(Constant)	11.12	14.324	–	0.776	0.238
x_1_	Gender	0.199	2.932	0.003	0.068	0.646
x_2_	Age	0.037	0.119	0.012	0.311	0.456
x_3_	Marital status	3.008	7.367	0.016	0.408	0.383
x_4_	Length of stay	4.636	0.308	0.544	15.054	< 0.001
x_5_	Discharge	1.489	3.451	0.016	0.432	0.366
x_6_	Type of insurance	1.508	0.453	0.212	2.375	0.002

^*^P-value Correlation is significant at the 0.05 level, B unstandardized coefficients, Std. error standard error

## Discussion

The present study aimed to determine OOP payment in the COVID-19 reference hospital in southern Iran. According to the current study’s findings, the OOP rate of hospitalized patients accounted for 8.89% of the total direct costs, with an average cost of 167.69 US $ per patient. Chua et al. averaged OOP for COVID-19 in 1,465 patients (according to the PharMetrics Plus for Academics database) over 90 days after discharge. They reported values from $534 for patients with private insurance and 680 US $ for patients covered by Medicare Advantage Plans [39]. Furthermore, Eisenberg et al. (2020) assessed the financial risk for COVID-19-like respiratory hospitalizations in the United States. They reported that the average OOP for patients admitted with respiratory infection in the two groups of consumer-directed health plans (CDHPs) and traditional plans were 1961 US $ and 1653 US $, respectively [40]. The findings of this study and previous studies indicate that COVID-19 disease has been associated with financial pressures imposed on patients because of hospitalization and the payment of a part of the treatment costs in the form of OOP. In addition to the need to pay for treatment, COVID-19 seems to have imposed financial pressure on patients by depriving them of some sources of income [41].

According to the results of this study, 57.12% of the OOP rates for the hospitalized patients were associated with pharmaceutical services, and the pharmaceutical cost had the largest share of OOP. Darab et al. in their study on the economic burden of COVID-19 in a reference hospital in southern Iran, reported that the costs of medicines and consumables devices with 28% of the total OOP were ranked second [42]. Medicines are one of the main expenditure items in the treatment of diseases, which, in line with the present study, are reported as the largest share of direct costs in many studies [43, 44].

The present results revealed a significant relationship between the OOP of hospitalized patients and the length of stay. Patients’ OOP rates also increased with the increasing length of stay. Hajizadeh and Nghiem conducted a study to promote an understanding of inequality and determinants of the OOP payments and exorbitant health costs for hospital services in Iran using national health services statistics. They concluded that the length of stay were significantly associated with patients’ OOP and increased likelihood of exorbitant health costs [31]. Taheri et al. studied all discharged patients (above 12,000 patients) from a teaching hospital in the United States. They found out that reducing the patients’ length of stay by a day decreases the total care cost by an average of three percent or less [45]. Riascos and Serna in Colombia conducted a study to predict the patients’ length of stay and estimate its impact on the health costs of about one million persons who had received at least one service during the last year and had not changed their insurance company during 2009–2011. They showed that the patients’ long stay in hospitals was costly for service providers, insurers, and patients due to increased demand for health services and the likelihood of serious risks during the stay [46]. Increasing the number of hospitalization days would also affect the cost and quality of the provided care [47]. Prolonged hospitalization increases the use of limited resources and the depreciation of hospital functions [48]. On the other hand, with an increase in length of stay, the costs of patients and the hospital increase, and the patients’ recovery and rehabilitation time also increase, thereby increasing costs [49]. To decrease costs, the length of unnecessary stay for patients should be reduced; hence, different interventions such as discharge planning, care strategies, periodic audits to detect and act against care delays, the use of checklists to plan admissions, the detection of motivated reference physicians can be implemented. Such interventions not only can reduce the hospitalization cost but also the incidence of infections and limited access to resources available to patients can be decreased [50, 51].

The present study suggested a statistically significant relationship between OOP for the COVID-19 patients and the discharge method in the death group. In their research, El-Khatib et al. reported a positive relationship between the mortality rate of the COVID-19 disease and patients’ OOP [52]. Yang et al. also revealed that the OOP rate in patients increases significantly with approaching death [53]. The significant relationship between OOP and mortality rates can be because patients with worse conditions need to spend more resources to regain their health, increasing hospital costs and patient payments.

Finally, the findings also indicated a significant relationship between patients' OOP and the type of insurance program. In their study in China, You and Kobayashi reported that some types of insurance programs were associated with increased patients’ OOP rates [54]. In Mexico, Galárraga et al. found out that voluntary public insurance for the unemployed and self-employed individuals was associated with a 54% decrease in catastrophic health expenditures [55]. Finkelstein and McKnight (2008) also reported a 40 percent decrease in patients’ OOP following the introduction of Medicare in the United States in 1965 [56].

## Conclusions

The present study reported the hospitalized patients’ OOP rate of 8.89%. The findings of this study confirm the urgent need to make decisions and implement effective interventions against COVID-19 by controlling risk factors and using other countries’ successful experiences and international organizations’ recommendations to reduce the prevalence and consequently the economic burden of the disease on patients and expand the role of insurance organizations.

The main limitation of the present study was that the OOP rate was estimated based on the documents and financial records of patients registered in HIS. Some patients, however, may have other OOP payments, such as informal payments and payments for drugs not available in the hospital. Such costs are likely to be reflected in official records.

## Data Availability

The datasets used and/or analyzed during the current study are available from the corresponding author on reasonable request.
